# Cofactors of drug hypersensitivity—A monocenter retrospective analysis

**DOI:** 10.3389/falgy.2022.1097977

**Published:** 2023-01-06

**Authors:** Johanna Kühl, Björn Bergh, Matthias Laudes, Silke Szymczak, Guido Heine

**Affiliations:** ^1^ Allergy Division, Department of Dermatology and Allergy, University Hospital Schleswig-Holstein, Kiel, Germany; ^2^ Institute of Medical Informatics and Statistics, Kiel University and University Hospital Schleswig-Holstein, Kiel, Germany; ^3^ Institute of Diabetes and Clinical Metabolic Research, University Medical Center Schleswig-Holstein, Campus Kiel, Kiel, Germany; ^4^ Institute of Medical Biometry and Statistics, University of Lübeck and University Medical Center Schleswig-Holstein, Campus Lübeck, Lübeck, Germany

**Keywords:** drug allergy, drug hypersensitivity reaction, antibiotic, beta-lactam, nonopioid analgesic, cofactor, multiple logistic regression

## Abstract

**Background:**

Drug hypersensitivity reactions (DHRs) are major medical problems that influence the treatment of patients by both under- and overdiagnosis. Still, little is known about the role of predisposing or protecting cofactors of DHR.

**Objective:**

This study aims to determine drug-specific cofactors in patients with DHR.

**Methods:**

Retrospective file chart analysis of inpatients with suspected DHR in our department between 2015 and 2020 was performed. Descriptive statistics and multiple logistic regression were conducted for the estimation and statistical interference.

**Results:**

DHRs were suspected in 393 patients with 678 culprit drugs. In 183 cases, drug hypersensitivities were confirmed, mostly against nonopioid analgesic drugs and antibiotics. Multiple logistic regression analysis identified a positive association of antibiotic hypersensitivity with obesity [odds ratio (OR) 5.75, average marginal effect (AME) +24.4%] and age and a negative association with arterial hypertension, female sex, elevated immunoglobulin E (IgE), and allergic rhinitis. Hypersensitivity to nonopioid analgesics was associated with atopic dermatitis (OR 10.28, AME +28.5%), elevated IgE, and arterial hypertension.

**Conclusions:**

Drug-specific cofactors of DHR include obesity for antibiotics and atopic dermatitis for nonopioid analgesics, the knowledge of which may improve the risk calculation for drug provocation tests.

## Introduction

Drug hypersensitivity reactions (DHRs) are major medical problems that influence the treatment of patients by both under- and overdiagnosis (1). In clinical practice, limited resources often lead to a diagnosis based on history alone (2). This entails the risk of overdiagnosis since only a few cases of suspected DHR are confirmed as allergic reactions by extensive diagnostics (2). The use of second-line medication may result in reduced disease control, drug-specific side effects, and/or higher costs (3, 4). Also, in pain treatment, only a limited number of alternative drugs to nonopioid analgesics exist. Overall epidemiologic data on DHR are sparse (1). Frequent drugs eliciting DHR are antibiotics (mainly beta-lactams) and nonopioid analgesics (mainly nonsteroidal anti-inflammatory drugs (NSAIDs), paracetamol (acetaminophen), and metamizole (dipyrone)) (1, 5, 6). The underlying mechanisms of DHR are complex and include allergic (immune-mediated) and pseudoallergic (nonimmune-mediated) reactions. In addition, multifactorial mechanisms may lead to DHR, but little is known about the contributing cofactors. For anaphylaxis, the existence of comorbidities and concomitant, so-called cofactors is suggested by the analysis of large registry databases, such as the network of severe allergic reactions (NORA), identifying age, psychological burden, vigorous physical exercise, beta-blockers, ACE inhibitors, and systemic mastocytosis (7). However, the data on DHR are limited. Although skin testing and the diagnostic strategies *in vitro* have improved during the past few decades, especially regarding type I allergies through the use of recombinant allergens, drug provocation test (DPT) still serves as a gold standard to diagnose or exclude DHR. New cost-effective diagnostic measures such as a risk-stratification before testing could improve the diagnostic procedure of DHR, which may include the knowledge of significant cofactors.

Here, we performed a monocentric, retrospective drug-chart analysis. Our data from a representative cohort strongly suggest the existence of drug-specific cofactors.

## Methods

The retrospective data from inpatients with suspected DHR of the Allergy Division at the Department of Dermatology, Venerology and Allergology, University Hospital Schleswig-Holstein Kiel, visiting between January 2015 and October 2020 were analyzed in an anonymized fashion, as approved by the local ethics committee, Medical Faculty of the Christian-Albrechts-University, Kiel, Germany (D541/20). The cases had been retrieved with the ICD-10 codes Z88, T78.4, and T88.7. The variables of interest were categorized (Supplementary Table S1). For each suspected drug, one case was defined; hence, multiple cases resulted from patients with multiple culprit drugs. An allergic case was defined by the diagnosis stated in the final medical report, based on positive history, skin test result (prick, intracutaneous, patch), serum-specific immunoglobulin E (IgE), and/or exposure test, also considering close structural homology. Reactions were divided by the time between drug intake and reaction into immediate (<1 h), intermediate (1–12 h), late (>12 h), and unknown. Descriptive data were depicted in numbers and percentages or as medians with an interquartile range (IQR) for categorical or quantitative variables, respectively. Software “R” version 1.2.5033 was used for all statistical calculations. The level of significance was considered at *α* = 5%.

To identify significant comorbidities and concomitant factors (defined here as cofactors) of a drug hypersensitivity confirmation while accounting for confounding effects, a multiple logistic regression analysis was conducted with the outcome variable DHR confirmed/ruled out after an inductive bivariate analysis (see Supplementary Methods). To have a high diagnostic accuracy, only cases with proven or excluded drug hypersensitivity, as shown by DPT, were included in this analysis. Additionally, due to the sufficiently high positive predictive value of beta-lactam skin tests (8, 9), beta-lactam cases according to the clinical reaction and positive skin test were also included. A separate logistic regression analysis was performed for the drug classes nonopioid analgesics and antibiotics for cases with complete characteristics. Hence, we analyzed cofactors by comparing confirmed vs. excluded drug hypersensitivity cases of one drug class. This allowed for a drug-specific cofactor analysis and reduced possible confounding effects. To assure independence of observations, only one case per patient was considered. If in a patient, multiple DHRs were suspected and one was confirmed, this case was chosen; if none or more than one were confirmed, a random case was selected. The independent variables are shown in Supplementary Table S1.

Systematic backward selection *via* the Akaike information criterion (AIC) was used for model selection. Nonsignificant variables were reintroduced into the model if they strongly elevated the explanatory value of the model and seemed medically relevant. The variables asthma, atopic dermatitis, allergic rhinitis, and serum IgE were considered as possible confounders, and therefore, all were included in the model if selected *via* AIC. The goodness-of-fit of the computed model was measured by the Hosmer–Lemeshow test, and Tjur's R2 was employed to analyze the explanatory and predictive power. The odds ratios (ORs) were calculated, and additionally, the average marginal effects (AMEs) (10) were provided. The latter displays the average effect of a change in the explanatory variable (see Supplementary Methods).

## Results

**Figure 1:**
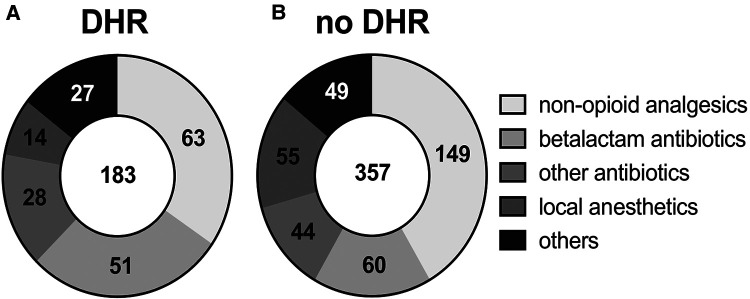
Drug classes in drug hypersensitivity. Diagrams depict the proportion of drug classes and number of cases of (**A**) confirmed and (**B**) excluded DHR.

We identified 393 patients with suspected drug hypersensitivity against 678 culprit drugs that fulfilled the analysis criteria (Supplementary Figure S1). Of those, 295 were female (75.1%), 97 were male (24.7%), and 1 unknown (0.2%). The median age was 53 years (IQR 26; 14–87 years) and the median body mass index (BMI) was 26.1 kg/m^2^ (IQR 7.4; 18.0–53.3 kg/m^2^, detailed patients’ characteristics in Supplementary Table S2). The frequencies of patients with normal serum IgE (78.5% < 100 kU/L) are in the range with epidemiologic findings of atopy (11). The prevalence of reported diseases was mostly consistent with the overall prevalence in Germany, e.g., similar to the prevalence of the general population (3.5%), 3.8% of the study cohort had atopic dermatitis (12). However, asthma (15.4% of the study cohort), allergic rhinitis (29.5%), and other self-reported hypersensitivities were overrepresented compared to the prevalence of asthma (8.6%) and allergic rhinitis (14.8%) in the German population (12). Obesity was overrepresented in the cohort presenting with a suspected antibiotics allergy (28.0%) compared to the entire study cohort (23.8%, in concordance with the German population) (13) and the cohort presenting with a nonopioid analgesics hypersensitivity (20.4%). Drug hypersensitivity was confirmed in 183 cases (27%), mostly nonopioid analgesics and antibiotics, and excluded in 357 cases (52.7%), see Figure 1 and Supplementary Tables S3–S5. Most antibiotics were beta-lactam antibiotics (Supplementary Table S3). From all nonopioid analgesics, paracetamol showed a low frequency of confirmed reactions (7.14%) compared to all other nonopioid analgesics (24.5%, Supplementary Table S3). Hypersensitivities to local anesthetics, although frequently suspected, were largely not confirmed (Figure 1). The diagnosis of a drug hypersensitivity was made through the clinical history (confirmed: 57, excluded 11 by differential diagnosis), DPT (confirmed: 51, excluded: 335), skin test (confirmed: 68, excluded: 11), serum-specific IgE (confirmed: 1), and structural homology (confirmed: 6).

Next, a multiple logistic regression model was applied to identify potential cofactors of the most frequent DHR. Antibiotic hypersensitivity was positively associated with obesity (OR 5.75, AME +24.4%), beta-lactams (OR 16.78, AME +39.3%), and with age (OR 1.05, AME +0.7%) (Table 1). Negative associations for antibiotic DHR were observed for elevated serum IgE 100–200 kU/L (OR 0.05, AME −32.1%), arterial hypertension (OR 0.13, AME −28.1%), allergic rhinitis (OR 0.20, AME −22.5%), and female sex (OR 0.19, AME −22.9%) (Table 1). Diagnosed nonopioid analgesic hypersensitivity was significantly associated with atopic dermatitis (OR 10.28, AME +28.5%), elevated IgE 200–1,000 kU/L (OR 4.95, AME +25.1%), and arterial hypertension (OR 4.53, AME +18.5%) (Table 1). Thus, these analyses show drug-specific differences in cofactors.

**Table 1:** Identified cofactors associated with antibiotic hypersensitivity diagnosis.

Predictors—antibiotics	OR (95% CI)^a^	AME (95% CI)^a^	*p*-value^b^
Sex	**0.19** (0.04 to 0.72)	**−0.229** (−0.404 to −0.054)	**0** **.** **020**
Age	**1.05** (1.02 to 1.09)	**0.007** (0.003 to 0.011)	**0** **.** **006**
Allergic asthma	0.93 (0.08 to 8.13)	−0.009 (−0.324 to 0.305)	0.953
Atopic dermatitis	3.78 (0.31 to 50.71)	0.185 (−0.145 to 0.515)	0.283
Allergic rhinitis	**0.20** (0.04 to 0.82)	**−0.225** (−0.416 to −0.033)	**0** **.** **034**
Total IgE 100–200 kU/L	**0.05** (0 to 0.51)	**−0.321** (−0.476 to −0.165)	**0** **.** **028**
Total IgE 200–1,000 kU/L	2.83 (0.37 to 26.9)	0.161 (−0.158 to 0.48)	0.325
Total IgE >1,000 kU/L	3.86 (0.3 to 113.01)	0.207 (−0.212 to 0.627)	0.343
Beta-lactams^c^	**16.78** (4.05 to 99.46)	**0.393** (0.23 to 0.557)	**<0** **.** **001**
Hypertension	**0.13** (0.02 to 0.68)	**−0.281** (−0.5 to −0.062)	**0** **.** **023**
Obesity	**5.75** (1.45 to 27.33)	**0.244** (0.063 to 0.425)	**0** **.** **018**
**Predictors—nonopioid analgesics**			
Age	1.03 (0.99 to 1.07)	0.003 (−0.001 to 0.008)	0.168
Allergic asthma	0.55 (0.07 to 3.03)	−0.073 (−0.295 to 0.148)	0.518
Atopic dermatitis	**10.28** (1.12 to 118.78)	**0.285** (0.027 to 0.543)	**0** **.** **040**
Allergic rhinitis	0.83 (0.21 to 2.95)	−0.023 (−0.183 to 0.137)	0.780
Total IgE 100–200 kU/L	1.75 (0.19 to 10.49)	0.071 (−0.199 to 0.341)	0.569
Total IgE 200–1,000 kU/L	**4.95** (1.01 to 25.24)	**0.251** (0.028 to 0.529)	**0** **.** **048**
Total IgE >1,000 kU/L	3.86 (0.09 to 171.63)	0.203 (−0.445 to 0.851)	0.449
Food allergy	3.00 (0.81 to 10.78)	0.135 (−0.015 to 0.285)	0.089
Hypertension	**4.53** (1.11 to 20.74)	**0.185** (0.015 to 0.355)	**0** **.** **041**
Comorbidities	0.78 (0.51 to 1.07)	−0.031 (−0.075 to 0.014)	0.181
Chronic treatment	0.81 (0.60 to 1.04)	−0.025 (−0.057 to 0.007)	0.135

Logit models. Observations Antibiotics 98, Tjur’s R2 0.419, Hosmer–Lemeshow test p-value 0.66. Observations nonopioid analgesics 126, Tjur’s R2 0.210, Hosmer–Lemeshow test p-value 0.67.

^a^ 95% confidence intervals in association of logit-model-identified cofactors with positive antibiotic or nonopioid analgesics hypersensitivity diagnosis.

^b^ Calculated within a multiple logistic regression model; total IgE categories were compared to normal values (20–100 kU/L).

^c^ Compared to non-beta-lactam antibiotics.

AME, average marginal effects. The Bold letters indicate statistically significant values.

## Discussion

Still, the knowledge of cofactors associated with DHR is limited but required. In this retrospective analysis, the outcome of detailed diagnostics to confirm or exclude DHR was associated with comorbidities and other cofactors. Our cohort is representative according to baseline characteristics with more female patients, age, BMI, total serum IgE, and also the investigated culprit drugs, the frequencies of confirmed DHR of mostly nonopioid analgesics, beta-lactams, and other antibiotics (14–16), and excluded DHR with safe reintroduction (2, 15, 16). This provides the prerequisite for identifying cofactors in further investigations.

The data suggest a higher frequency of obesity in patients presenting with suspected antibiotics hypersensitivity than for nonopioid analgesics, as well as an association of confirmed hypersensitivity to antibiotics with obesity. Our data support the recent observation that obesity is more frequent in patients with confirmed drug allergy (total *n* = 84 with 34 positive DHR, 93% were antibiotics) (17) and penicillin allergy (18). Whether this results from more frequent use of antibiotics and thus the probability of sensitization or whether obese patients present rather after stronger and certain reactions is not known but would explain the data. The overrepresentation of obese patients in the antibiotics cohort could be connected to obese-related drug indications, e.g., skin infections; however, the separate analysis of the antibiotics cohort indicates that the association between obesity and the diagnostic outcome has other underlying reasons. Overall, obesity as such does not appear to be the dominant risk factor for developing antibiotic allergy, suggesting additional factors are required. Accordingly, in the large cross-sectional FoCus cohort of well-defined patients with obesity (19), the prevalence of self-reported antibiotic hypersensitivity between obese and nonobese patients was not significantly different [83 of 1,800 probands, 5.45% (39/716 in BMI > 30 kg/m^2^), 4.00% (43/1,075 in BMI < 30 kg/m^2^, chi-square test *p* = 0.18, unpublished results]. In addition, the observed inverse association between antibiotic hypersensitivity and arterial hypertension could be attributed to infection-driven increased hypersensitivity mimicking allergy, e.g., arterial hypertension and following, e.g., flushing, erythema, or dyspnea, which would be a promising target of future research, e.g., assessing serum tryptase concentrations after reaction compared to baseline levels or histologic assessment of exanthema in a standardized fashion.

Relevant cofactors of DHR to nonopioid analgesics were atopic dermatitis, elevated serum IgE, and arterial hypertension. Whether direct mechanisms are responsible or indirect, e.g., by increased analgesics-consumption to treat hypertension-induced symptoms, will be clarified by future studies.

The observed negative association of the female sex with proven antibiotic hypersensitivity [not positive as observed earlier (20)] as well as females not being prone to anaphylaxis as such (21) support the hypothesis that women use health services more frequently with reactions that have a lower probability of confirmation, e.g., by a high health awareness. In line, males also appear to have a higher risk for severe anaphylaxis reactions (7). The data showing the association of arterial hypertension and elevated serum IgE in an opposing manner for nonopioid analgesics and antibiotics support that drug class-specific diagnostics are important to avoid false-negative results.

In this study, the atopic comorbidities allergic rhinitis and allergic asthma are overrepresented in suspected DHR (12). In line with the finding that allergic rhinitis is a strong negative predictor for antibiotic hypersensitivity, this may be the result of increased awareness for allergic reactions in atopic individuals or/and a higher allergy work-up rate of low-risk reactions (e.g., penicillin allergy during childhood) due to existing access to an allergy department. Our data emphasize the importance of conducting drug provocation tests in these individuals to avoid a false diagnosis of drug hypersensitivity and facilitate delabeling.

The limitation of this study is the retrospective design and resulting missing data on cofactors not acquired in a standardized manner that limited the number of cases and statistical modeling and subsequent analysis of rare drugs. Particularly, the designation of type I or type IV reactions was not clearly recorded in the file. This resulted in many cases from (lacking) information provided by the patient, especially if the presentation in the clinic was months after the reaction. The exact documentation of the last treatment dose before the reaction in hours in addition to the treatment duration in days should be recorded as well as the exact symptoms for every patient to avoid data losses. The inquiry of the infection cause in a standardized manner together with the antibiotic drug hypersensitivity reaction may improve the research regarding additional factors beyond HIV or Epstein-Barr-Virus (EBV) and exanthema on cotrimoxazole or amoxicillin. This may be overcome by a prospective design and multicentric setting, which may also allow us to analyze the impact of very rare comorbidities such as mast cell disorders.

In conclusion, our data from a representative cohort suggest that drug-specific cofactors are associated with antibiotic and nonopioid analgesics hypersensitivity, which may improve the risk calculation for drug exposure testing and future mathematical risk prediction models as well as offer targets for further mechanistic research.

## Data Availability

The original contributions presented in the study are included in the article/Supplementary Material, further inquiries can be directed to the corresponding author.
