# Supplementary material for: Cofactors of drug hypersensitivity—A monocenter retrospective analysis
Source: Front Allergy. 2023 Jan 6;3:1097977. doi: 10.3389/falgy.2022.1097977 (PMC9854260; doi:10.3389/falgy.2022.1097977)
Supplement: Supplementary file 1 [file Datasheet1.pdf]

Cofactors of Drug Hypersensitivity – a mono-center retrospective analysis

Johanna Kühl, Björn Bergh, Matthias Laudes, Silke Szymczak, Guido Heine

Supplementary Methods

To assess different characteristics in allergic and non-allergic patients, non-normally distributed quantitative variables were compared using the Mann-Whitney-Wilcoxon test and categorical variables using the Pearson's chi-square test (Fisher's exact test for small sample sizes) with Yates continuity correction. Not included in the final analysis were 11 cases identified as NSAID intolerance reactions (1.6%, dose-dependent reactions in a DPT) and the remaining 127 cases not finally clarified (mostly due to lost-to-follow-up and substitute testing, also because of severe primary reaction and the patient's fear of a possible test reaction despite detailed information of the procedure or other medical reasons, eFigure 1). Anaphylaxis or severe cutaneous drug reactions were no exclusion criterion per se. If the patients showed a dose-independent reaction, a nonopioid hypersensitivity was diagnosed (independent of cross-reactive NSAIDs) and included into the analysis.

Considering all drugs, few associations of cofactors were determined in this inductive bivariate analysis (data not shown), however, these were not confirmed by the consecutive and more reliable logistic regression analysis. Most likely due to the small patient numbers per drug and confounding variables, a distinct effect of a drug-specific cofactor would be below the detection threshold. Thus, cofactors were analyzed in detail for patients with reactions to the most frequent drug classes, i.e. antibiotics and nonopioid analgesics, via multiple logistic regression analysis.

For the interpretation of the results, two methods were chosen. Additionally to the routinely used odds ratio (OR), the average marginal effects (AMEs) are presented to allow for a more intuitive interpretation as average change of probability of a DHR confirmation in case of exposure to a binary variable or small changes in a continuous variable, respectively.

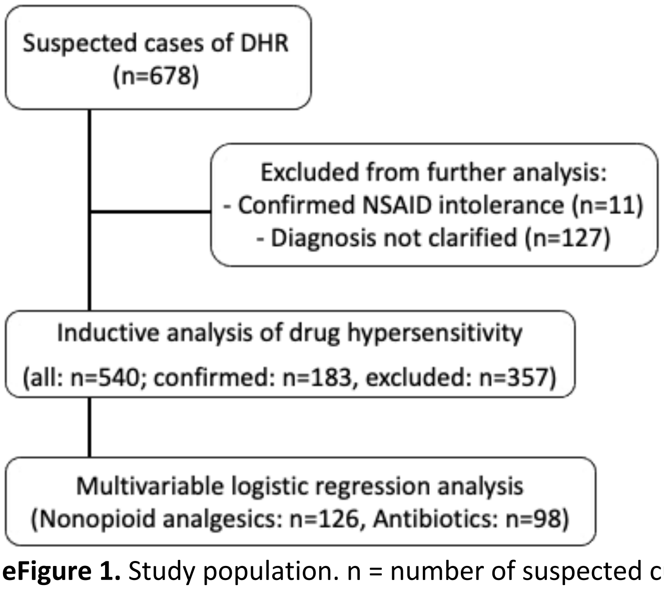

eFigure 1. Study population. n = number of suspected culprit drugs, here defined as cases.

eTable 1. Definitions of variables.

Variable	Definition	Final recording
Drug hypersensitivity	The final diagnosis as stated in the medical letter	No = 0, Yes = 1, "unclear", "confirmed NSAID intolerance". The latter two were excluded in further analysis
Age	Mean age at time of visiting the Allergy Department in Kiel	Years
Sex	Sex of patient	Male = 0, Female = 1
BMI	Weight/(height/100)^2	Numeric
Pre-existing conditions	Number of pre-existing conditions	Numeric
Comorbidities	Number of comorbidities = number of pre-existing conditions minus the "status post conditions"	Numeric
Chronic treatment	Number of chronic pharmacological treatment taken by the patient	Numeric
Simultaneously taken drugs	Number of simultaneously taken drugs at time of reaction	Numeric
Atopic dermatitis	Present or history of atopic dermatitis	No = 0, Yes = 1
Asthma	Present or history of asthma	No = 0, Yes = 1
Cardiovascular risk factors	Number of cardiovascular risk factors	Numeric
Hypertension	Diagnosed arterial hypertension	No = 0, Yes = 1
Beta blockers	Reported intake of beta blockers	No = 0, Yes = 1
ACE inhibitor	Reported intake of ACE inhibitors	No = 0, Yes = 1
Diabetes	Diagnosed diabetes mellitus type 2	No = 0, Yes = 1
Allergic rhinitis	Self-reported allergic rhinitis	No = 0, Yes = 1
Food allergy	Self-reported food allergy or hypersensitivity	No = 0, Yes = 1
Contact allergy	Self-reported contact allergy	No = 0, Yes = 1
Other drug hypersensitivities	Other self-reported drug hypersensitivities that are not currently suspected	No = 0, Yes = 1
Other hypersensitivities	Any self-reported hypersensitivity in patient independent of the type (e.g. allergic rhinitis, food allergy, contact allergy, other drug hypersensitivities, bee and wasp venom allergy)	No = 0, Yes = 1
Obesity	BMI > 30 kg/m^2	No = 0, Yes = 1
Overweight	BMI > 25 kg/m^2	No = 0, Yes = 1
Total-IgE	Categorized value of total-IgE	0 = Norm: 20 - 100 kU/l, 1 = 100-200 kU/l, 2 = 200 - 1000 kU/l, 3 = > 1000 kU/l
Latency	Categorized time from the last drug exposure to onset of symptoms	1 = Immediate: < 1 hour, 2 = Intermediate: 1- 12 hours, 3 = Late: over 12 hours, 4 = Unknown
Betalactam Dummy	Drug class to which the culprit drug studied belongs is a betalactam antibiotic	No = 0, Yes = 1

Route of Administration	Way in which the culprit drug was administered	1= Oral 2 = Intravenous, 3 = Subcutaneous, 4 = Other, 5 = Unknown
Systemic Anaphylaxis	Anaphylaxis Grade II-IV, definition and classification according to Ring and Messmer[1]	No = 0, Yes = 1, if presence of urticaria, flushing or angioedema with at least one of the following: shortness of breath, hypotension, tachycardia, shock, circulatory dysregulation, faecal incontinence, urinary incontinence, loss of consciousness, laryngeal edema, cardiopulmonary resuscitation.
Year of Visit	Year of the first inpatient treatment	Year
Skin Test	Results of skin tests (include prick, intracutaneous, and patch tests)	Unclear = 0, Negative = 1, Positive = 2
Drug Provocation Test	Status of drug provocation test: Was a drug provocation test performed for the suspected culprit drug?	No = 0, Yes = 1

Total-IgE categories were chosen based on the existing literature [2-5]. The category 1 was introduced in order to pick up some of the possible effect of concomitant atopic diseases. In 60% of cases, the latency period was not recorded in detail. In the logistic regression analysis we considered the variable "latency period" (categorized into <1h, 1-12h, >12h, unknown) or alternatively in type I (<12h), type IV (>12h), and unknown. No significance or increase in explanatory value of the model was found, therefore, the variable was not included into the final model. The variable hypertension and the drugs ACE inhibitors and beta-blockers showed perfect multicollinearity and thus the latter were not included into the analysis.

eTable 2. Distribution of putative cofactors in the study cohort. Subdivided by diagnosis.

Characteristic	Allergy	No Allergy	Unclear	Total
Female	95 (70.4%)	156 (76.5%)	41 (82%)	292 (75.1%)
Male	40 (29.6%)	48 (23.5%)	9 (18%)	97 (24.9%)
Atopic dermatitis	6 (4.4%)	7 (3.4%)	2 (4%)	15 (3.8%)
Asthma	18 (13.3%)	31 (15.1%)	11 (22%)	60 (15.4%)
Allergic rhinitis	35 (25.9%)	65 (31.7%)	15 (30%)	115 (29.5%)
Food allergy	28 (20.7%)	39 (19%)	15 (30%)	82 (21%)
Contact allergy	26 (19.3%)	45 (22%)	9 (18%)	80 (20.5%)
Other drug hypersensitivities	37 (27.4%)	54 (26.3%)	15 (30%)	106 (27.2%)
Other hypersensitivities	86 (63.7%)	128 (62.4%)	35 (70%)	249 (63.8%)
Hypertension	40 (29.6%)	51 (24.9%)	12 (24%)	103 (26.4%)
ACE inhibitors	10 (7.4%)	9 (4.4%)	5 (10%)	24 (6.2%)
Beta blockers	14 (10.4%)	24 (11.7%)	7 (14%)	45 (11.5%)
Diabetes	10 (7.4%)	14 (6.8%)	3 (6%)	27 (6.9%)
Obesity	39 (28.9%)	42 (20.5%)	12 (24%)	93 (23.8%)
Overweight	70 (51.9%)	101 (49.3%)	24 (48%)	195 (50%)

This overview includes the prevalence of binary variables.
Confirmed NSAID intolerances were excluded.

eTable 3. Suspected culprit drugs and confirmed drug allergy.

Drug Class	Suspected Culprit Drug	Cases	Confirmed
Nonopioid analgesics	Acetylsalicylic Acid	48	9
	Diclofenac	30	13
	Ibuprofen	80	18
	Dipyrone/Metamizole	63	19
	Paracetamol/Acetaminophen	28	2
	Others	8	2
	Total	*257*	*63*
Beta-lactam antibiotics	Amoxicillin (AX)	38	12
	AX-Clavulanicacid	5	1
	Ampicillin	5	3
	Ampicillin-Sulbactam	11	4
	Cephalosporins	46	16
	Meropenem	1	1
	Penicillin V/G	31	14
	Total	*137*	*51*
Other Antibiotics	Clindamycin	29	13
	Cotrimoxazole	14	5
	Doxycycline	8	0
	Quinolones	13	5
	Macrolides	14	3
	Others	11	2
	Total	*89*	*28*
Local Anesthetics	Articaine	33	3
	Bupivacaine	5	3
	Lidocaine	13	3
	Mepivacaine	11	3
	Prilocaine	12	1
	Others	13	1
	Total	*87*	*14*
Others	Other Drugs	69	16
	Narcotics	12	5
	Opioids	15	4
	Proton-pump inhibitors	12	2
	Total	*108*	*27*
Total	-	678	183

eTable 4. Indication for antibiotic therapy.

Indication for antibiotic therapy	No.	% of all
Unknown	61	27%
Operation	15	6.6%
Dental work	11	4.9%
Flu-like infection	13	5.8%
Other	8	3.5%
Post operation	7	3.1%
Urinary tract infection	15	6.6%
Upper respiratory tract infection	12	5.3%
Tonsillitis	13	5.8%
Bronchitis	11	4.9%
Pyelonephritis	6	2.7%
Inflammation of teeth/gum	6	2.7%
EBV infection (misdiagnosis)	4	1.8%
Prophylaxis	3	1.3%
Abscess	3	1.3%
Infection of the skin (not specified)	5	2.2%
Cystitis	5	2.2%
Erysipelas	4	1.8%
Inflammation of the rooth canal	3	1.3%
Pneumonia	3	1.3%
Diverticulitis	2	0.9%
Helicobacter pylori eradication therapy	3	1.3%
Eradication of resistant germs	3	1.3%
Erythema migrans	2	0.9%
Sepsis	2	0.9%
Wound infection	2	0.9%
Lyme arthritis	2	0.9%
Otitis media	2	0.9%

eTable 5. Indication for nonopioid analgesic therapy.

Indication for analgesic therapy	No.	% of all
Unknown	88	34.2%
Pain	103	40.1%
Operation	23	8.9%
Dental work	5	1.9%
Flu-like infection	16	6.2%
Other	8	3.1%
Post operation	10	3.9%
Pyelonephritis	2	0.8%
Tendinitis	2	0.8%

Supporting References:

1. Ring J, Messmer K. Incidence and severity of anaphylactoid reactions to colloid volume substitutes. *Lancet.* 1977;1:466-469.

2. Moreno EM, Moreno V, Laffond E, Gracia-Bara MT, Munoz-Bellido FJ, Macias EM, et al. Usefulness of an Artificial Neural Network in the Prediction of beta-Lactam Allergy. *J Allergy Clin Immunol Pract.* 2020;8:2974-2982 e2971.

3. Wong CY, Yeh KW, Huang JL, Su KW, Tsai MH, Hua MC, et al. Longitudinal analysis of total serum IgE levels with allergen sensitization and atopic diseases in early childhood. *Sci Rep.* 2020;10:21278.

4. Sunyer J, Anto JM, Castellsague J, Soriano JB, Roca J. Total serum IgE is associated with asthma independently of specific IgE levels. The Spanish Group of the European Study of Asthma. *Eur Respir J.* 1996;9:1880-1884.

5. Tay TR, Bosco J, Aumann H, O'Hehir R, Hew M. Elevated total serum immunoglobulin E (>1000 IU/mL): implications? *Intern Med J.* 2016;46:846-849.
